# The use of the Washington Group on Disability Statistics questionnaires to identify hearing disability: a systematic review

**DOI:** 10.1590/2317-1782/20212020328

**Published:** 2022-01-12

**Authors:** Jennifer Dantas Moreno, Larissa Hellen Viégas Bennett, Silvia Ferrite

**Affiliations:** 1 Departamento de Fonoaudiologia, Universidade Federal da Bahia – UFBA - Salvador (BA), Brasil.; 2 College of Health Professions, School of Audiology, Pacific University - Hillsboro (OR), USA.

**Keywords:** International Classification of Functioning Disability and Health, Hearing, Hearing Loss, Disability Evaluation, Surveys and Questionnaires

## Abstract

**Purpose:**

To investigate the use of instruments from the Washington Group on Disability Statistics (WG) to obtain data on hearing disability (HD).

**Research strategies:**

We conducted searches in the PubMed, Scopus, Science Direct, Web of Science, Lilacs databases and the grey literature. The software “The State of the Art through Systematic Review” and “Mendeley” were used to assist in the bibliographic reference organization, selection, and storage.

**Selection criteria:**

we followed the guidelines proposed by the “Preferred Reporting Items for Systematic Reviews and Meta-Analysis” and we selected studies that met the following inclusion criteria: written in English or Portuguese, within the period of 2001 to 2017 and have used the WG hearing disability question.

**Data analysis:**

The variables analyzed into the studies were: WG module, country and year of data collection, sample size and composition, objective of the study, publication journal, HD estimate of prevalence and accuracy measures.

**Results:**

Sixty-five studies are included in the review, conducted with data from 30 countries. The WG Short Set of question was the most often used. Hearing disability prevalence ranged from 0.2 to 2.3% and only three studies estimated the accuracy of the instrument to identify HD.

**Conclusion:**

The hearing disability question of WG has been used worldwide and mainly in developing countries. The short variation in the estimated prevalence measurements within studies seems favorable to the WG's goal of generate estimates that allow international comparison. However, the shortage of validity studies indicates the need for further investigations with this purpose.

## INTRODUCTION

Through its Classification of Functioning, Disability and Health (ICF), the World Health Organization (WHO) recognizes that disability is not limited to a deviation from a standard norm, in other words it is not an individual attribute, but rather a complex interaction between people with impairments and contextual factors. Disability is considered an umbrella term for impairments and, above all, encompasses the negative aspects of this interaction, including behaviour and environmental barriers that limit activities and the individual’s full, effective, and egalitarian participation in society^(1-3)^. In 2018, disabling hearing loss, defined by the WHO as hearing thresholds in the better ear below 40dB in adults and 30dB in children, affected 466 million people (6.1% of the global population), with an estimated projection of 630 million in 2030 and 900 million in 2050, in the absence of effective prevention activities^(4)^.

Hearing loss can have several negative impacts on the individual. When considering the four components defined by the ICF – body function, body structure, activity and participation, and contextual factors – hearing loss starts to be observed not only through the focus of damage to the hearing organ, but also by taking account of the multidimensional experience of individuals with hearing loss. In this sense, the functionality approach provided progress in terms of the possibility of more reliably characterizing the impact of disability on the subject’s life, although gaps remain in the development of adequate tools that enable the collection of data regarding hearing disabilities, preferably in order to provide international comparisons^(3,5)^.

In 2001, the Washington Group on Disability Statistics (WG) was set up with support from the United Nations (UN) to develop tools to obtain data about disabilities and their impact on the individual’s quality of life. When the study was conducted, the WG contained three questionnaires: the WG Short Set on Functioning (WG-SS), the WG Extended Set on Functioning (WG-EF) and the WG/UNICEF Module on Child Functioning (WG/UNICEF). In all three questionnaires, the question regarding hearing disability considered the degree of difficulty and the use of sound amplification devices^(5-7)^.

The use of these questionnaires has been recorded on censuses in several countries, although there are few studies about the results obtained and the instrument’s validity in the countries where it has been applied^(8,9)^. Identifying hearing disability is complex, since it involves several factors, including environmental, social and cultural ones, hampering differentiation between the health condition and its impact on functionality^(10,11)^. It is, however, necessary, as part of the process to guarantee inclusion and the right to the dignified participation of all individuals in society.

The development of tools that enable the collection of data that identifies hearing disability in a way that allows for international comparisons is important for the generation of epidemiological evidence to support the planning and evaluation of public policies and intervention strategies.

## OBJECTIVE

The study objective was to investigate the use of questionnaires from the Washington Group on Disability Statistics to obtain data about hearing disability.

### RESEARCH STRATEGY

We undertook a systematic review of the quantitative and qualitative literature, using descriptive analysis, based on a selection of scientific articles, dissertations, theses and official publications which applied the question about hearing disability developed by the WG, and were published between January 2001 and December 2017. The initial year was chosen because 2001 was the year the WG was created.

In May 2018, we surveyed the literature by searching the PubMed (International Biomedical and Lifesciences), Lilacs (Scientific Health Information from Latin America and the Caribbean countries), Science Direct and Web of Science databases. Our descriptors were identified through the MeSH - Medical Subject Headings. In addition to these descriptors we added terms and expressions collected through previous readings of articles on this subject. The English language terms and descriptors were: “Washington group”; “short set”; “extended set”; “Washington questionnaire”; “disability module”; “health survey”; “hearing”; “hearing loss”. The combination of terms and descriptors for each of the databases can be found in Appendix 1. As well as these databases, we also conducted searches in the grey literature by accessing the thesis database of the Coordination for the Improvement of Higher Education Personnel (*Coordenação de Aperfeiçoamento de Pessoal de Nível Superior*: CAPES) and the Google Scholar (https://scholar.google.com) search engine. We used the “Washington Group” AND “hearing” Boolean string in these searches. Recently, the Cochrane Handbook for Systematic Reviews of Interventions and the Institute of Medicine's Standards for Systematic Review have begun to recommend the incorporation of different data sources in systematic analyses^(12)^ to include those not published in academic sources.

To support the storage of abstracts, and the organization and selection of bibliographical references obtained from the databases, we used free StArt (State of the Art through Systematic Review) software, a computer tool for bibliographical management specifically developed to carry out systematic reviews^(13)^. Using this tool, we identified duplicate works recovered from different databases, excluded any duplicates, to ensure we only considered one version. Mendeley free software^(14)^ was used to store complete documents.

### SELECTION CRITERIA

The process for drafting and conducting the systematic review followed the guidelines of the Prisma (Preferred Reporting Items for Systematic Reviews and Meta-Analyses)^(15)^ declaration. The PRISMA recommendations consist of a checklist containing 27 items and a flowchart with four stages. Once identified through the searches (stage 1), selection (stage 2) consisted of a systematic reading of the publications’ titles, abstracts and descriptors. We then excluded those works that did not fulfil the inclusion criteria (stage 3). Finally, we conducted a complete reading of the selected documents and included those that fulfilled all the eligibility criteria for review (stage 4). The inclusion criteria to select works were: original scientific articles, official documents, dissertations or theses, published in English or Portuguese between January 2001 and December 2017; and which asked the WG question about hearing disability to obtain data. Stages 2 to 4 were conducted independently by two reviewers, both Speech-Language-Hearing professionals. At any stage, cases of disagreement between the reviewers were resolved through consensus.

### DATA ANALYSIS

We extracted data from the selected works with the support of a data extraction form and used Excel 2016 to construct the tables. The following data was extracted: objective, method, results and conclusion, journal and its respective impact factor, country and year the question was applied, participant demographic characteristics, the estimated prevalence of identified hearing disability and accuracy measures.

The variables for analysis were defined as: journal; year/time of application of WG module; study objective, categorized as (a) associates disability with other factors, (b) estimates disability prevalence, (c) undertakes comparison between instruments and (d) instrument validation; WG module used; country where the question was applied; sample size and composition; estimated prevalence of hearing disability; accuracy measures.

## RESULTS

**Figure 1:**
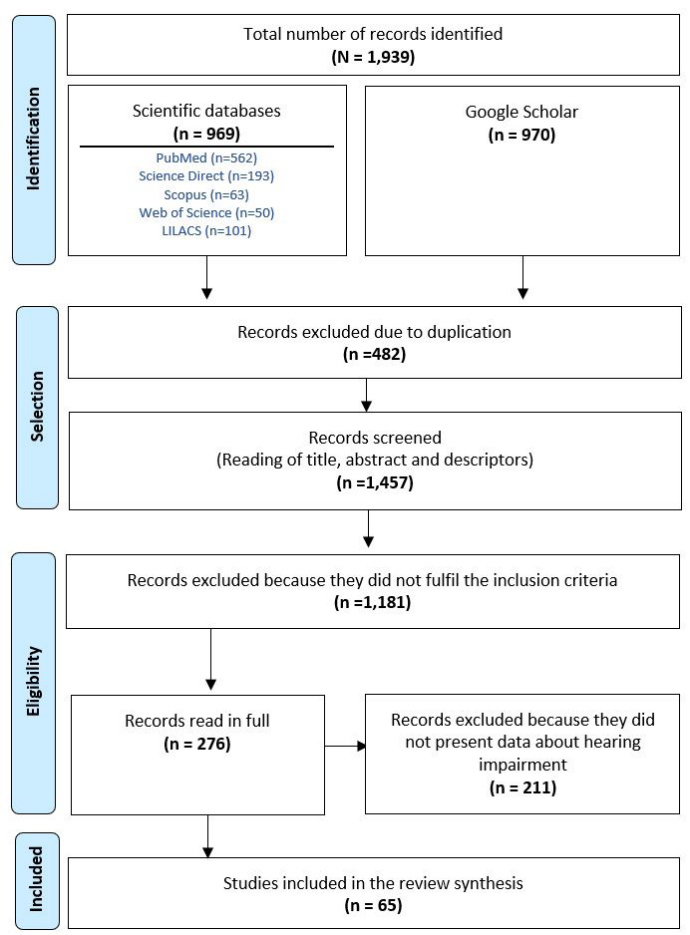
Flowchart mapping the search and selection of scientific works for a systematic review of the use of the Washington Group on Disability Statistics hearing disability question, using PRISMA* criteria

In all, 1,939 works were identified in the database searches and on Google Scholar. Of these, 969 were articles from scientific databases and 970 works were collected through a search of the grey literature. Works eliminated due to database duplication totalled 482. The 1,457 remaining works were screened as part of the first stage of the selection process. During this stage, following a reading and analysis of titles, abstracts and key words, 1,181 texts were excluded because they did not meet the established eligibility criteria. The 276 remaining works were read in full with the aid of the standardized information extraction form, of these, 211 works were excluded, since they did not include data about applying the hearing disability question. Sixty-five studies were therefore selected for synthesis within the systematic review, as seen in the Figure 1 flowchart.

*Preferred Reporting Items for Systematic Reviews and Meta-Analyses

Most of these studies were scientific articles published in journals with a high impact factor and h-index (Table 1). These indices, considered scientific coefficients, quantify the repercussion journals have within the academic environment. This finding suggests that the theme of functionality and/or use of WG questions for hearing disability is visible within the scientific community and well received by quality periodicals.

**Table 1:** Characteristics of studies selected in the review of literature about the use of the Washington Group on Disability Statistics’ tools to identify hearing disability, 2017

Variables	N=65	%
**Type of Study**		
Article	53	81.5
Report/Official Document	12	18.5
**Journal** * **(IF; H)**		
Plos One (IF:1.16; H:241)	7	10.8
Bio Med Central (IF:1.34; H:103)	6	9.2
Disability and Rehabilitation (IF:0.8; H:92)	4	6.2
ALTER - European Journal of Disability Research (IF:0.3; H:11)	3	4.6
BMJ Open (IF: 1.01; H:24)	3	4.6
Disability and Health Journal (IF:1.95; H:24)	3	4.6
Population Health Metrics (IF:1.95; H:40)	2	3.1
Tropical Medicine & International Health (IF: 1.73; H:97)	2	3.1
World Development (IF: 2.12; H:140)	2	3.1
Others	33	50.8
**Study Year**		
2001 - 2004	3	4.6
2005 - 2008	14	21.5
2009 - 2012	26	40.0
2013 - 2016	22	33.8
**Objectives**		
To estimate prevalence	16	24.6
To investigate factors associated with disability	40	61.5
Instrument validation studies	1	1.5
Comparison between instruments	8	12.3
**Module**		
WG Short Set	53	81.5
WG Extended Set	4	6.2
WG/UNICEF Children Disability	6	9.2
WG Extended Set and WG/UNICEF Children Disability	2	3.1

* Journals from which more than one study was selected for the review

Caption: IF = Impact Factor; H = h-index. Source: Scimago Journal & Country Rank^(16)^

We observed a significant growth between 2009 and 2012 in the number of studies that applied WG instruments, with a similar level maintained over the following four years. This finding is consistent with the recommendation made at the 2008 UN Convention on the Rights of People with Disabilities to use questions developed by the WG in demographic censuses and population surveys^(17)^.

In fact, the main aim of the WG was to create a tool capable of generating comparable data about disability at global level, which is easy to incorporate into censuses in different countries^(17)^. In 2002, during the group’s first meeting, there was agreement about prioritizing the development of the WG Short Set, an instrument that analyses six functional domains or basic actions: vision, hearing, mobility, cognition, self-care and communication. In the English version, the question the group published about hearing is as follows: *Do you have difficulty hearing, even if using a hearing aid?*; versions are presented in several languages, with the recommendation that they be subject to a specific protocol in order to avoid inconsistencies and/or erroneous interpretations and to increase the validity and use of collected data. The response categories in the module vary according to degree of difficulty thus: No, no difficulty, Yes, some difficulty, Yes, a lot of difficulty and Cannot do it at all. In order to determine the status of disability in censuses, the WG recommends that disability be considered when the responses meet one of the following parameters: a) at least some difficulty in two domains b) and/or a lot of difficulty or worse in one domain^(18)^. The WG Extended Set on Functioning (WG-EF), created in 2005, was also based on the ICF and considers aspects of difficulty, that is, it seeks to establish the manifestation of limitations, the onset of disability, its duration, cause, and the practice of activities^(6)^. The group's most recent questionnaire is the child module. Created in 2011 in partnership with UNICEF, the WG/UNICEF Module on Child Functioning and Disability seeks to identify children with disabilities in the domains of vision, hearing, mobility, communication/understanding, learning, relationships and the ability to play.

We observed that the WG Short Set questionnaire was the more frequently used and was adopted by 53 of the 65 studies, probably because of its practical nature and ease of application^(19,20)^. In 2014, the UN identified that 35 countries had used the short module in national censuses to identify disability or impairment^(21)^. For our review, we only selected studies that recorded the WG question about hearing disability. We therefore identified 30 countries, with the African regions and Southeast Asia the most represented (Table 2). In addition to selection criteria, other factors may have contributed to the fact that not all countries that used the WG questions in censuses were represented in this review’s documents. For example, publication language (we only considered records in Portuguese/English) and availability (we only collected files indexed in digital media).

**Table 2:** Distribution of studies that used one of the Washington Group modules, by health region and country (N=65)

REGION*	**COUNTRY**	**NUMBER OF STUDIES**
**AFRICA**	Cameroon	7
	South Africa	6
	Uganda	5
	Zambia	3
	Ethiopia	2
	Burkina Faso	1
	Ghana	1
	Lesotho	1
	Kenya	1
	Sierra Leone	1
	Tanzania	1
**THE AMERICAS**	Mexico	3
	United States	2
	Peru	2
	Guyana	1
	Haiti	1
**EUROPE**	Portugal	2
**WESTERN MEDITERRANEAN**	Palestine	1
**WESTERN PACIFIC**	Vietnam	5
	Fiji	2
	Philippines	2
	Cambodia	1
	Malaysia	1
	Mongolia	1
**SOUTHEAST ASIA**	India	7
	Bangladesh	6
	Indonesia	2
	Nepal	2
	Myanmar	1
	Sri Lanka	1

* Regions defined according to the World Health Organization

Despite this limitation, this study identified a significant number of records from countries that apply the WG questionnaire in order to estimate prevalence of hearing disability. However, most of the selected studies opted to use the questionnaires to measure and define the variable of hearing disability, in order to analyse how certain factors are related to the disability^(5,10,20,22-58)^.

In the context of a shortage of human and technological resources in developing countries, the use of low cost instruments to estimate hearing disabilities is one alternative^(59)^. Fujiura et al.^(60)^ confirmed that the way different nations understand disability and impairment is reflected in the method they use to identify them. At the time, developing countries generally used questions in their population research that related to disability based on the identification of the presence or lack of physical limitations, while developed countries were preoccupied with identifying aspects related to functionality and disability.

Recognition of the concept and the identification of disability supports an understanding of processes related to the experience of disability, since it considers aspects of interaction between an individual (with a health problem) and the contextual and environmental factors they experience^(61)^. By considering multiple interactions, any discussion of functionality and disability based on this theoretical model favours a board understanding of different levels and the severity of an individual’s limitations. According to Schneider^(62)^, using the term “difficulty” in the WG modules allows for the identification of people who would not otherwise be captured by the term “disability”, for example the elderly; and the identification of people with more severe difficulties, in other words, those who recognize that they are disabled, who respond with the answers “a lot of difficulty” or “cannot do it at all.” This more inclusive WG strategy to identify disabilities therefore keeps track of estimated disability and also enables access to those who experience mild or moderate difficulty. In this context, the assessment of disability is a fundamental approach to monitoring and modulating strategies, at both individual and collective level, aimed at advances in functionality, that is, at reducing disability despite the impairment, helping to reduce inequality in social participation^(9)^. From this perspective, the skills of people with disabilities may expand, and they may experience improvements in their well-being and freedom, thereby expanding their rights^(63)^.

Of the 65 studies identified, the majority were conducted with large samples, varying from 30 to 513,219 individuals, with a median of 3,140. Of the 17 studies focused on estimating the prevalence of hearing disability and/or accuracy measures, 65% investigated samples of more than 4,000 individuals. In population studies, principally conducted to generate estimates of health indicators, having a large sample provides a representative population^(64)^. Given that one of the WG’s main objectives is to support the dissemination of questions about functionality in censuses, national studies, and other applications, the studies analysed in this review demonstrate the feasibility of using the question to investigate hearing disability on a large scale. We note other factors that demonstrate the WG questionnaire’s large-scale application, including the fact that it is a rapid assessment instrument with multiple choice answers, and specific training is not required to apply it. In this context, Sprunt et al.^(65)^ expanded the instruments’ feasibility by using key informants, such as teachers, given that, if the structure to hold clinical tests is limited, these professionals can be the main responders for the identification of disabilities in children. Similarly, Khandaker et al.^(34)^ argued that, as well as being effective and low-cost, the key informant method provides high sensitivity for the identification of children with disabilities. Other researchers have applied the questionnaire with carers^(7,65-69)^ and old people^(20,25,70,71)^.

Regarding estimates of hearing disability prevalence (Table 3), we observed that when the cut-off point recommended by the WG (“a lot of difficulty”) was applied, there was little variation between the estimates obtained (0.2-2.3%). However, when using the “some difficulty” cut-off point, the estimates in these studies differed considerably, from 1.4% to 15.9%. The “some difficulty” option appears to provide a broader and more diverse interpretation for the level of severity experienced by the individual, favouring the identification of impairments but hindering the comparability of estimates between populations. We note that 16 studies (24.6%) made adaptations to the modules^(73,76,77)^, in particular to the response options, which is not recommended by the WG, and compromises comparative analysis between studies^(19,66,69-82)^.

**Table 3:** Studies which contained data about the prevalence of hearing disability according to type of Washington Group module used

**Study**	**Country**	**Year**	**Population**	**Prevalence of hearing disability**				
				Binary response (yes) for difficulty	Most of the time	Some difficulty or worse	A lot of difficulty or worse*	**Cannot do at all**
**SHORT SET**								
Ahmad et al.^(72)^	Malaysia	2015	19,959 individuals 18-30 years (35%) to > 60 years (11.2%)			5.5%		
Ramachandra et al.^(73)^	India	2014	4,000 adults 18-28 years (22.2%) to >70 years (7.6%)		**1.60%**			
Ferrite et al.^(74)^	Cameroon	2013	4,104 individuals 0-9 years (34.7%) to >80 years (2.5%)			14.10%	**1.10%**	
Danquah et al.^(75)^	Haiti	2012	3,132 individuals 0-16 years (21%) to >16 years (79%)			1.4%	**0.20%**	**0.04%**
Islam et al.^(76)^	Bangladesh	2012	3,104 individuals 30-35 years (7.7%) to > 65 years (14.7%)	**16.50%**				
				12.6% M				
				18.5% F				
Wandera et al.^(71)^	Uganda	2010	2,628 **old people (>50 years)**			10.7% M	1.5%M	
						15.9% F	2.2% F	
Tareque et al.^(70)^	Bangladesh	2010	4,176 **old people (>60 years)**			**10.32%**	**2.16%**	**0.26%**
						9% M	2% M	0.19% M
						11.7% F	2.31% F	0.34% F
Marella et al.^(77)^	Bangladesh	2010	2,315 adults 18 -24 years (23.8%) to > 50 years		**2.30%**			
Bachani et al.^(19)^	Uganda	2009	5,247 individuals 0-14 years (39%) to >60 years (6%)			2.6% M		
						3.2% F		
Loeb et al.^(78)^	Zambia	2006	28,010 individuals (all ages)			3.70%	**2.30%**	**0.50%**
Seager and Tamasane^(49)^	South Africa	2005	942 street-dwelling adults and 305 children (12-73 years). Answered by key informants			5.9% (child)	**3.0%(children)**	**0%(children)**
						10.4% (adult)	**1.7%(adults)**	**1.7%(adults)**
EXTENDED SET								
Moniruzzaman et al.^(79)^	Bangladesh	2009	37,030 individuals (all ages)				**1.00%**	**0.40%**
WG/UNICEF								
Rojas-Martínez et al.^(69)^	Mexico	2015	5,010 children (2-4 years)				**0.16%**	
							0.22%M	
							0.1% F	
Geda et al.^(52)^	Ethiopia	2014	21,572 children (0-14 years)				**1.94%**	
							2.14%M	
							1.71% F	

* Washington Group recommended cut-off point

Caption: F = Female; M = Male

The studies in the review aimed at analysing factors associated with hearing disability were able, amongst other factors, to identify a lower level of country development, aging, lower socio-economic conditions (family income, employment and the granting of benefits) and gender^(25,28,30,42,58)^. People in more vulnerable situations more frequently referred to having hearing difficulties^(37,46,48)^. In self-reported questionnaires, the prevalence of hearing disability was more frequently reported by women than men^(22,35,66,70)^. The diversity of these association studies appears to ratify and reinforce the use of the WG module to measure and define the variable of hearing disability in different contexts.

Few studies estimated accuracy measures (Table 4). Sprunt et al.^(65)^ recommend that any investigation of validity should analyse each domain separately and that different cut-off points for the degree of difficulty should be considered. When analysing the question about hearing in isolation, for the “some difficulty or worse” cut-off point, sensitivity and specificity values of 67% and 88% were found respectively, while for “a lot of difficulty or worse” these values were 22% and 99.6% respectively^(74)^. The sensitivity and specificity measures for the hearing disability question in the WG child module were close to those obtained for the short set. It is worth noting that better accuracy was observed when the instrument was used with a teacher^(65)^. The Mactaggart et al.^(10)^ study of a population of children and adults in Cameroon and India demonstrated that the “a lot of difficulty” cut-off point missed a large proportion of individuals with disabilities, usually between the different domains, and that this pattern is accentuated when considering the vision and hearing domains, where functional limitations are less likely to be self-reported.

**Table 4:** Distribution of studies that presented accuracy measures for hearing disability obtained from the Washington Group hearing module compared to pure tone audiometry

**Study**	**Questionnaire/Instrument**	**Population/Domain analysed**		**Sensitivity**		**Specificity**		**Positive Predictive Value**		**Negative Predictive Value**	
				Some difficulty or worse	A lot of difficulty or worse	Some difficulty or worse	A lot of difficulty or worse	Some difficulty or worse	A lot of difficulty or worse	Some difficulty or worse	A lot of difficulty or worse
Sprunt et al.^(**^65^**)^	WG/UNICEF Hearing question	(N=472) Children ages 5-15 - Special and Inclusive Education Schools, Fiji	a*	78%	41%	88%	97%	--	--	--	--
			b*	72%	50%	95%	99%	--	--	--	--
Loeb et al.^(78)^	Complete Short Set Module	(N= ﻿5751) All Ages - Rural and Urban Families, Zambia		--	97,7%	--	98,5%	--	--	--	--
Ferrite et al.^(74)^	Short Set Hearing question	(N= 3567) All Ages – Rural Families, Cameroon		67%	22%	88%	99,6%	16%	65%	99%	97%

* Questionnaire applied to: (a) Parents; (b) Teachers

When applying the WG as a screening instrument for functionality at population level, the adoption of a cut-off point of analysis should therefore take account of its implications, while the results obtained for each cut-off point should be assessed parsimoniously.

## CONCLUSION

The instruments of the Washington Group on Disability Statistics are used to identify hearing disability at global level, particularly in developing countries. The small variation between prevalence measures appears to be a favourable factor for the international comparability of estimates, which is one of its main strengths.

However, in this review, we observed that the cut-off point and other methodological differences may compromise the comparability of estimates; for this reason we recommend using the instrument in the format proposed by the WG and, wherever possible, presenting results that consider the two most common cut-off points: “a lot of difficulty” or worse; “some difficulty” or worse. More studies of the instrument's validity are also required, particularly to investigate the different cut-off points.

The positive points and advantages of using the instrument to measure hearing disability, such as ease of application and understanding, favour its dissemination for use in different scientific investigations, which go beyond censuses and prevalence studies.

## Appendix 1 Search strategy by database

**Table:**

**PUBMED**	**SCOPUS**	**SCIENCE DIRECT**	**WEB OF SCIENCE**	**LILACS**
Search (((“washington group”) OR (((“short set” OR “extended set”)) AND question))) AND (((((“washington group”) OR (((“short set” OR “extended set”)) AND question)) OR (((“washington questionnaire”) OR module disability) OR “do you have difficulty”))) AND (((((((((domain) OR function) OR disability) OR impairment) OR disorder) OR “health survey”[MeSH Terms]) OR hearing) OR “hearing loss”) OR difficulty))	((TITLE-ABSKEY (“washington group”)) OR (((TITLE-ABS KEY (“short set”) OR TITLE-ABS-KEY (“extended set”))) AND (TITLE-ABS-KEY (question))) OR ((TITLE-ABS-KEY (“Washington question”)OR TITLE-ABS-KEY (“Washington module disability”) OR TITLE-ABS-KEY ({do you have difficulty})))) AND ((TITLE-ABS KEY (function) OR TITLE-ABS-KEY (domain) OR TITLE-ABS-KEY (disability) OR TITLE-ABS-KEY (impairment) OR TITLE-ABS-KEY (disorder) OR TITLE-ABS-KEY (“health survey”) OR TITLE-ABS-KEY (hearing) OR TITLE-ABS-KEY (“hearing loss”) OR TITLE-ABS-KEY (difficulty))	(((“washington group W/2 disability”) or (“washington group”)) OR ((({short set?}) or ({extended set?})) AND ({question?}))) AND (((hearing) OR (“hearing loss”) OR (difficulty)) AND ((function) OR (domain) OR (disability) OR (impairment) OR (disorder)))	Topic: (function) *OR* Topic: (domain) *OR* Topic: (difficulty) *OR* Topic: (hearing) *OR* Topic: (“hearing loss”) *OR* Topic: (“health survey”) *OR* Topic: (impairment) *OR* Topic: (disorder*) *OR* Topic: (disability)*AND* Topic: (“Washington question*”) *OR* Topic: (“do you have difficulty”) *OR* Topic: (“washington module”) *OR* (“short set” OR “extended set”) *OR* (“Washington group”)	(tw:(“washington group” OR “short set” OR “extended set”)) AND (tw:((tw:(domain)) OR (tw:(function)) OR (tw:(disability)) OR (tw:(impairment)) OR (tw:(disorder)) OR (mh:(“health survey”)) OR (tw:(hearing)) OR (tw:(“hearing loss”)) OR (tw:(difficulty))))
