# Supplementary material for: The use of the Washington Group on Disability Statistics questionnaires to identify hearing disability: a systematic review
Source: Codas. 2022 Jan 12;34(2):e20200328. doi: 10.1590/2317-1782/20212020328 (PMC9769417; doi:10.1590/2317-1782/20212020328)
Supplement: Translation

# Translation: A utilização dos questionários do *Washington Group on Disability Statistics* para identificação da incapacidade auditiva: uma revisão sistemática

**Authors:** Jennifer Dantas Moreno, Larissa Hellen Viégas Bennett, Silvia Ferrite

**Affiliations:** 1 Departamento de Fonoaudiologia, Universidade Federal da Bahia – UFBA - Salvador (BA), Brasil.; 2 College of Health Professions, School of Audiology, Pacific University - Hillsboro (OR), USA.

**Keywords:** Classificação Internacional de Funcionalidade Incapacidade e Saúde, Audição, Perda Auditiva, Avaliação da Deficiência, Inquéritos e Questionários

## Abstract

**Objetivo:**

Investigar a aplicação dos instrumentos do *Washington Group on Disability Statistics* (WG) para obtenção de dados sobre incapacidade auditiva (IA).

**Estratégia de pesquisa:**

Foram conduzidas buscas nas bases de dados *PubMed, Scopus, Science Direct, Web of Science e Lilacs,* e na literatura cinzenta. Os softwares *State of the Art through Systematic Review* e *Mendeley* foram utilizados como ferramentas para organização, seleção e armazenamento dos documentos.

**Critérios de seleção:**

Seguiram-se orientações propostas pela *Preferred Reporting Items for Systematic Reviews and Meta-Analyses.* De acordo com critérios de inclusão, foram selecionados estudos publicados em inglês ou português, entre 2001 e 2017, que aplicaram a pergunta sobre IA do WG.

**Análise dos dados:**

Foram definidas como variáveis de análise: módulo WG utilizado, país e ano da coleta de dados, tamanho e composição da amostra, objetivo do estudo, periódico de publicação, estimativa da prevalência de IA e medidas de acurácia.

**Resultados:**

Foram 65 os estudos incluídos, conduzidos com dados de 30 países. O módulo curto do WG foi mais comumente utilizado. As estimativas de prevalência da IA variaram de 0,2 a 2,3%, e apenas três estudos estimaram a acurácia do instrumento para sua identificação.

**Conclusão:**

O emprego do instrumento do WG, módulo de incapacidade auditiva, tem ocorrido em nível mundial, principalmente em países em desenvolvimento. A pequena variação entre as medidas de prevalência estimadas pelos estudos parece ser favorável ao objetivo do WG em gerar estimativas que permitam comparação internacional. No entanto, a escassez de estudos de validade indica a necessidade de mais investigações.

## INTRODUÇÃO

A Organização Mundial da Saúde (OMS), através da Classificação de Funcionalidade, Incapacidade e Saúde (CIF) reconhece que a deficiência não se resume a um desvio do padrão normativo, ou seja, não é um atributo de pessoa, mas uma complexa interação entre as pessoas com impedimentos e fatores contextuais. Incapacidade é considerado o termo genérico para deficiências e engloba sobretudo os aspectos negativos dessa interação, incluindo as barreiras comportamentais e ambientais que limitam as atividades e participação plena, eficaz e igualitária do indivíduo na sociedade^(1-3)^. A perda auditiva incapacitante, definida pela OMS como limiares de audibilidade piores que 40dB para adultos e 30 dB para crianças na melhor orelha, afetava 466 milhões de pessoas (6,1% da população mundial) em 2018, com projeção estimada de 630 milhões em 2030 e 900 milhões em 2050, na ausência de ações efetivas de prevenção^(4)^.

A perda de audição pode acarretar diversos efeitos negativos ao indivíduo. Ao considerar os quatros componentes definidos pela CIF – função do corpo, estrutura do corpo, atividade e participação e fatores contextuais – a perda auditiva passa a ser observada não apenas sob o foco da lesão do órgão da audição, mas considerando também a experiência multidimensional vivenciada por indivíduos com perda auditiva. Neste sentido, a abordagem da funcionalidade trouxe avanços no que se refere à possibilidade de caracterizar de maneira mais fidedigna o impacto da deficiência sobre a vida do sujeito, embora ainda haja lacunas no desenvolvimento de ferramentas adequadas que possibilitem a obtenção de dados referentes à incapacidade auditiva, preferencialmente de modo a permitir comparações internacionais^(3,5)^

O *Washington Group on Disabilities Statistics* (WG) foi criado em 2001, apoiado pela Organização das Nações Unidas (ONU), com o objetivo de desenvolver ferramentas para obtenção de dados acerca das incapacidades e do seu impacto na qualidade de vida dos indivíduos. No período da realização deste estudo, o WG contava com três questionários: o *WG Short Set of Questions* (WG-SS), o *WG Extended Set of Questions on Functioning* (WG-EF) e o *UNICEF/WG Module on Children Functioning* (UNICEF/WG). Em todos eles, a pergunta sobre incapacidade auditiva considerava o grau de dificuldade e o uso do aparelho de amplificação sonora^(5-7)^.

A utilização destes questionários tem sido registrada nos censos de diversos países, apesar de ainda haver uma escassez de estudos sobre os resultados obtidos e a validade do instrumento nos países onde foram aplicados^(8,9)^. A identificação da incapacidade auditiva é complexa, pois envolve diversos fatores, como os ambientais, sociais e culturais, dificultando a distinção entre a condição de saúde e o seu impacto na funcionalidade^(10,11)^. Ela é, porém, necessária, como parte do processo para garantir a inclusão e os direitos à participação digna de todos os indivíduos na sociedade.

O desenvolvimento de ferramentas que viabilizem coletar dados que identifiquem as incapacidades auditivas, e ainda de modo que permitam comparações internacionais, é relevante para gerar evidências epidemiológicas que subsidiem o planejamento e a avaliação de políticas públicas e estratégias de intervenção.

## OBJETIVO

O objetivo deste estudo foi investigar a utilização dos questionários do *Washington Group on Disability Statistics* para obtenção de dados sobre incapacidade auditiva.

### ESTRATÉGIA DE BUSCA

Foi desenvolvida uma revisão sistemática de literatura, descritiva, de abordagem quantitativa e qualitativa, a partir da seleção de artigos científicos, dissertações, teses e publicações oficiais que utilizaram a pergunta sobre incapacidade auditiva desenvolvida pelo WG e que foram publicados no período de janeiro de 2001 a dezembro de 2017. Justifica-se a escolha do ano inicial pois 2001 foi o ano de criação do WG.

Foi conduzido levantamento da literatura em maio de 2018 por meio de buscas nas bases PubMed (Literatura Internacional de Ciências da Saúde), Lilacs (Literatura Latino-Americana e do Caribe em Ciências da Saúde), Science Direct, Web of Science. Os descritores utilizados na pesquisa foram identificados através dos *MeSH - Medical Subject Heading*. Além dos descritores, foram adicionados termos e expressões coletados através da leitura prévia de artigos relacionados ao assunto. Os termos e descritores, pesquisados no idioma inglês, foram*: “Washington group”; “short set”; “extended set”; “Washington questionnaire”; “module disability”; “health survey”; “hearing”; hearing loss”.* A combinação dos termos e descritores utilizados em cada base de dados encontra-se no Apêndice 1. Além das bases de dados, também foram conduzidas pesquisas na literatura cinzenta através do acesso ao banco de teses da Coordenação de Aperfeiçoamento de Pessoal de Nível Superior (Capes) e na plataforma de buscas Google Acadêmico (https://scholar.google.com). Foram utilizados nas buscas a cadeia booleana *“Washington Group”* AND *“hearing”*. Recentemente, o Manual Cochrane de Revisões Sistemáticas de Intervenções e o Instituto de Padrões de Medicina para Revisão Sistemática passaram a recomendar a incorporação de diferentes fontes de dados em análises sistemáticas^(12)^, desta forma, busca-se garantir o acesso a documentos formais que não são publicados em fontes acadêmicas.

Para auxiliar no armazenamento dos resumos, organização e seleção das referências bibliográficas obtidas nas bases de dados, foi utilizado o software livre e gratuito Start (*State of the Art through Systematic Review*), ferramenta computacional de gerenciamento bibliográfico desenvolvida especificamente para condução de revisões sistemáticas^(13)^. Com essa ferramenta, foram identificados os trabalhados em duplicidade recuperados nas diferentes bases, e excluídas as duplicatas sendo considerada apenas uma versão. O software livre e gratuito, Mendeley^(14)^ foi utilizado para o armazenamento dos documentos completos.

### CRITÉRIO DE SELEÇÃO

O processo de elaboração e condução da revisão sistemática seguiu as orientações da declaração Prisma – *Preferred Reporting Items for Systematic Reviews and Meta-Analyse* ^(15)^. As recomendações PRISMA consistem em um checklist com 27 itens e um fluxograma de quatro etapas. Uma vez identificados nas buscas (etapa 1), a seleção (etapa 2) consistiu na leitura sistemática dos títulos, resumos e descritores das publicações. Na sequência, foram excluídos os trabalhos que não atenderam aos critérios de inclusão (etapa 3). Por fim, realizou-se a leitura completa dos documentos selecionados, e foram incluídos na revisão os que atenderam a todos os critérios de elegibilidade (etapa 4). Os critérios de inclusão considerados na seleção dos trabalhos foram: artigos científicos originais, documentos oficiais, dissertações ou teses, publicados em inglês ou português, no período de janeiro de 2001 a dezembro de 2017; e que tenham utilizado a pergunta da WG sobre incapacidade auditiva para obtenção de dados. As etapas de 2 a 4 foram realizadas de maneira independente por dois revisores, ambos fonoaudiólogos. Os casos de discordância entre os revisores, em todas as etapas, foram resolvidos por consenso.

### ANÁLISE DOS DADOS

A extração de dados dos trabalhos selecionados foi realizada com o auxílio de uma ficha de extração. O programa Excel 2016 foi utilizado para construção das tabelas. Foram extraídos: objetivo, método, resultados e conclusão, periódico de publicação e seu respectivo fator de impacto, país e ano de aplicação da pergunta, características demográficas dos participantes do estudo, estimativa da prevalência de incapacidade auditiva identificada e medidas de acurácia.

As variáveis definidas para análise foram: periódico de publicação; ano/período de aplicação do módulo WG; objetivo do estudo, categorizado em (a) associar incapacidade com outros fatores, (b) estimar prevalência de incapacidade, (c) comparação entre os instrumentos e (d) validação do instrumento; módulo WG utilizado; país de aplicação da pergunta; tamanho e composição da amostra; estimativas de prevalência de incapacidade auditiva; medidas de acurácia.

## RESULTADOS

No total, foram identificados 1.939 trabalhos nas buscas nas bases de dados e no Google Acadêmico. Destes, 969 foram artigos oriundos das bases científicas e 970 trabalhos coletados através das buscas na literatura cinzenta. Os trabalhos eliminados por duplicidade nas bases de dados totalizaram 482. Os 1.457 trabalhos restantes participaram da triagem, primeira etapa do processo de seleção. Nessa etapa, após a leitura e análise dos títulos, resumos e palavras-chave, foram excluídos 1.181 textos visto que não se enquadravam nos critérios de elegibilidade estabelecidos. Os 276 trabalhos restantes, foram lidos na íntegra com o auxílio de ficha padronizada de extração de informação, e destes, 211 trabalhos foram excluídos, pois não apresentaram dados sobre a utilização da pergunta sobre incapacidade auditiva. Assim, 65 estudos foram selecionados para síntese da revisão sistemática conforme fluxograma apresentado na Figura 1.

**Figura 1:** Fluxograma do processo de busca e seleção dos trabalhos científicos para a revisão sistemática sobre o uso da pergunta do Washington Group on Disability Statistics sobre incapacidade auditiva de acordo com o critério PRISMA*

*Preferred Reporting Items for Systematic Reviews and Meta-Analyses

A maioria desses estudos foram artigos científicos publicados em revistas com fator de impacto e índice H altos (Tabela 1). Estes índices, considerados coeficientes científicos, quantificam a repercussão dos periódicos no meio acadêmico. Esse achado sugere que o tema da funcionalidade e/ou o uso das perguntas do WG para incapacidade auditiva tem tido visibilidade pela comunidade científica e boa receptividade por periódicos de qualidade.

**Tabela 1:** Características dos estudos selecionados na revisão de literatura sobre a utilização das ferramentas *do Washington Group on Disability Statistic*s para identificação da incapacidade auditiva, 2017

Variáveis	N=65	%
**Tipo de Estudo**		
Artigo	53	81,5
Relatório/Documento Oficial	12	18,5
**Periódico de Publicação** * **(FI; H)**		
Plos One (FI:1.16; H:241)	7	10,8
Bio Med Central (FI:1.34; H:103)	6	9,2
Disability and Rehabilitation (FI:0.8; H:92)	4	6,2
ALTER - European Journal of Disability Research (FI:0.3; H:11)	3	4,6
BMJ Open (FI: 1.01; H:24)	3	4,6
Disability and Health Journal (FI:1.95; H:24)	3	4,6
Population Health Metrics (FI:1.95; H:40)	2	3,1
Tropical Medicine & International Health (FI: 1.73; H:97)	2	3,1
World Development (FI: 2.12; H:140)	2	3,1
Outros	33	50,8
**Ano do Estudo**		
2001-2004	3	4,6
2005-2008	14	21,5
2009-2012	26	40,0
2013-2016	22	33,8
**Objetivos**		
Estimar Prevalência	16	24,6
Investigar fatores associados à incapacidade	40	61,5
Estudos de validação do instrumento	1	1,5
Comparação entre instrumentos	8	12,3
**Módulo**		
*WG Short Set*	53	81,5
*WG Extended Set*	4	6,2
*UNICEF/WG Children Disability*	6	9,2
*WG Extended Set e UNICEF/WG Children Disability*	2	3,1

* Periódicos que tiveram mais de um estudo selecionado na revisão

Legenda: FI = Fator de Impacto; H = Índice H. Fonte: Scimago Journal & Country Rank^(16)^

Nesta revisão, observou-se um crescimento significativo no número de estudos que aplicaram instrumentos do WG entre 2009 e 2012, mantendo patamar semelhante no quadriênio seguinte. Esse achado é coerente com a recomendação da “Convenção das Nações Unidas sobre os Direitos das Pessoas com Deficiências”, em 2008, para a utilização das perguntas desenvolvidas pelo WG nos censos demográficos e inquéritos populacionais^(17)^.

De fato, o WG tem como principal objetivo a criação de ferramentas capazes de gerar dados comparáveis sobre a incapacidade a nível mundial e de fácil incorporação aos censos dos diferentes países^(17)^. Durante o primeiro encontro do grupo, em 2002, foi acordado como prioridade o desenvolvimento do módulo curto-WG (*WG-Short Set of Questions*), instrumento que propõe a análise de seis domínios funcionais ou ações básicas: visão, audição, mobilidade, cognição, capacidade do autocuidado e comunicação. Na versão em inglês publicada pelo grupo, a pergunta sobre audição está organizada na seguinte estrutura: *“Do you have difficulty hearing, even if using a hearing aid?”*, para a qual são apresentadas versões em diversas línguas, com a recomendação de que sejam submetidas a um protocolo específico afim de evitar inconsistências e/ou interpretações errôneas e aumentar a validade e utilidade dos dados coletados. No módulo, as categorias de respostas variam por escala de gravidade em: não, nenhuma dificuldade; sim, alguma dificuldade; sim, muita dificuldade e; não é capaz de realizar. Para determinação de status de deficiência nos censos, o WG recomenda considerar como incapacidade quando as respostas atendem a um dos seguintes parâmetros: a) pelo menos alguma dificuldade em dois domínios b) e/ou muita dificuldade ou pior em um domínio^(18)^. O módulo extenso-WG (*WG-Extended Set of Questions on Functioning*), criado em 2005, também foi baseado na CIF, e considera as especificidades das dificuldades, isto é, procura estabelecer de que forma as limitações se manifestam, o início da incapacidade, sua duração, causa, e a prática de atividades^(6)^. O módulo infantil é o questionário mais recente do grupo. Criado em 2011, em uma parceria com a UNICEF, o *UNICEF/WG Module on Child Functioning and Disability* busca identificar crianças com impedimentos nos domínios da visão, audição, mobilidade, comunicação/compreensão, aprendizado, relacionamentos e habilidade de brincar.

Nesta revisão, observou-se que o questionário WG-*Short Set of Questions* foi o mais frequentemente utilizado, adotado por 53 dos 65 estudos, provavelmente pela praticidade e agilidade de aplicação^(19,20)^. Em 2014, as Nações Unidas identificaram que já eram 35 os países que utilizavam o módulo curto nos censos nacionais para identificação de deficiência e ou incapacidade^(21)^. Nesta revisão foram selecionados apenas os estudos que registraram a pergunta sobre a IA dos questionários do WG. Assim, foram identificados 30 países, sendo as regiões Africanas e do Sudeste Asiático aquelas com maior representatividade (Tabela 2). Além do critério de seleção, outros fatores podem ter contribuído para que nem todos os países que utilizaram as perguntas do WG nos censos estivessem representados com documentos contemplados nessa revisão. Por exemplo, o idioma de publicação (foram considerados apenas registros em português/inglês) e a disponibilidade (foram coletados apenas arquivos indexados no meio digital).

**Tabela 2:** Distribuição dos estudos que utilizaram um dos módulos do Washington Group de acordo com a região de saúde e país (N=65)

Região*	País	Número de estudos
AFRICANA	Camarões	7
	África do Sul	6
	Uganda	5
	Zâmbia	3
	Etiópia	2
	Burquina Fasso	1
	Gana	1
	Lesoto	1
	Quênia	1
	Serra Leoa	1
	Tanzânia	1
AMÉRICAS	México	3
	Estados Unidos	2
	Peru	2
	Guiana	1
	Haiti	1
EUROPEIA	Portugal	2
MEDITERRÂNICA ORIENTAL	Palestina	1
PACÍFICO OCIDENTAL	Vietnã	5
	Fiji	2
	Filipinas	2
	Cambodia	1
	Malásia	1
	Mongólia	1
SUDESTE ASIÁTICO	Índia	7
	Bangladesh	6
	Indonésia	2
	Nepal	2
	Myanmar	1
	Sri Lanka	1

* Regiões conforme definição da Organização Mundial da Saúde

Apesar desta limitação, este estudo identificou um número expressivo de registros de países que aplicaram o questionário da WG com o objetivo de estimar a prevalência da incapacidade auditiva. Embora a maioria dos estudos selecionados tenha optado por utilizar os questionários para mensurar e definir a variável incapacidade auditiva, tendo como objetivo analisar de que forma determinados fatores se relacionam com a incapacidade^(5,10,20,22-58)^.

No contexto de escassez de recursos humanos e tecnológicos em países em desenvolvimento, a utilização de instrumentos de baixo custo para estimativa de incapacidade auditiva é uma alternativa^(59)^. Fujiura et al.^(60)^ verificaram que o modo como as diferentes nações compreendem a deficiência/incapacidade se reflete no método que utilizam para sua identificação. À época, países em desenvolvimento, em geral, utilizavam em suas pesquisas populacionais perguntas relacionadas à deficiência a partir da identificação da presença ou não de impedimentos corporais, enquanto países desenvolvidos já se preocupavam em identificar aspectos relacionados à funcionalidade e incapacidade.

O reconhecimento do conceito e da identificação da incapacidade auxilia na compreensão dos processos relacionados com a vivência da deficiência, uma vez que considera os aspectos de interação entre um indivíduo (com problema de saúde) e os fatores contextuais e ambientais vivenciados por este^(61)^. Assim, a discussão sobre funcionalidade e incapacidade a partir desse modelo teórico, ao considerar as múltiplas interações, favorece uma compreensão ampliada sobre os diferentes níveis e sobre a gravidade das limitações do indivíduo. De acordo com ﻿Schneider^(62)^, o uso do termo “dificuldade” nos módulos da WG permite identificar pessoas que não seriam contadas usando o termo “deficiência”, por exemplo os idosos; além da identificação de pessoas com dificuldades mais severas, ou seja, aquelas auto reconhecidas com deficiência, que respondem nas categorias de “muita dificuldade” ou “não é capaz de realizar”. Portanto, essa estratégia mais inclusiva do WG para identificação da incapacidade mantém o registro da estimativa de deficiência além de permitir acesso àqueles que vivenciam dificuldades mais brandas ou moderadas. Neste contexto, a avaliação da incapacidade é abordagem fundamental para monitorar e modular estratégias, no nível individual ou coletivo, que visem progressos na funcionalidade, ou seja, redução da incapacidade apesar da deficiência, contribuindo para a diminuição da desigualdade na participação social^(9)^. Nessa perspectiva, pessoas com deficiência podem ter suas habilidades expandidas, além de melhorias no seu bem estar e liberdade favorecendo assim a ampliação dos seus direitos^(63)^.

Entre os 65 estudos identificados, predominaram aqueles realizados com grandes amostras, variando de 30 a 513.219 indivíduos, com mediana de 3.140. Dos 17 estudos que tinham como objetivo estimar a prevalência da incapacidade auditiva e/ou medidas de acurácia, 65% investigaram amostras com mais de 4.000 indivíduos. Em estudos de base populacional, utilizados principalmente para gerar estimativas de indicadores de saúde, uma amostra grande favorece a representatividade da população^(64)^. Considerando que um dos principais objetivos da WG é a disseminação do uso de questões sobre funcionalidade em censos, pesquisas nacionais, entre outras aplicabilidades, os estudos analisados nesta revisão demostram a factibilidade do uso da questão sobre incapacidade auditiva em grande escala. Destacam-se ainda outros fatores que corroboram a aplicação do questionário WG em larga escala, entre eles: ser um instrumento rápido, com resposta de múltipla escolha e que não exige formação específica do aplicador. Neste contexto, Sprunt et al.^(65)^ ampliam a factibilidade do uso desses instrumentos por informantes chaves, como por exemplo professores, uma vez que, em locais com estrutura limitada de realização de exames clínicos, esses profissionais podem ser os principais responsáveis pela identificação de deficiências em crianças. Similarmente, Khandaker et al.^(34)^ defendem que o método de informante-chave, além de ser eficaz e de baixo custo, apresenta alta sensibilidade para identificação de crianças com deficiência. Estudos também utilizaram o questionário aplicando-o a cuidadores^(7,65-69)^ e idosos^(20,25,70,71)^.

Em relação às estimativas de prevalência de incapacidade auditiva **(** **Tabela 3** **)**, observou-se que ao considerar o ponto de corte recomendado pelo WG (“muita dificuldade”) houve pouca variação entre as estimativas obtidas pelos estudos (0,2% – 2,3%). Entretanto, quando considerado o ponto de corte “alguma dificuldade”, as estimativas encontradas nos estudos diferiram consideravelmente, de 1,4% a 15,9%. A alternativa “alguma dificuldade” parece ofertar uma interpretação mais ampla e diversa sobre o nível de gravidade da experiência vivida pelo indivíduo, favorecendo a identificação de incapacidades, porém dificultando a comparabilidade da estimativa entre as populações. Destaca-se que 16 estudos (24,6%) realizaram adaptações nos módulos^(73,76,77)^, em especial nas alternativas de resposta, o que não é recomendado pela WG e compromete a análise comparativa entre estudos^(19,66,69-82)^.

**Tabela 3:** Estudos que apresentaram dados de prevalência de incapacidade auditiva de acordo com o tipo de módulo do Washington Group utilizado]

Estudo	País	Ano	População	Prevalência de incapacidade auditiva				
				Resposta binária (sim) para dificuldade	Na maior parte do tempo	Alguma dificuldade ou pior	Muita dificuldade ou pior*	**Não é capaz**
SHORT SET OF QUESTION								
Ahmad et al.^(72)^	Malásia	2015	19.959 indivíduos 18-30 anos (35%) a > 60 anos (11,2%)			5,5%		
Ramachandra et al.^(73)^	Índia	2014	4.000 adultos 18-28 anos (22,2%) a >70 anos (7,6%)		**1,60%**			
Ferrite et al.^(74)^	Camarões	2013	4.104 indivíduos 0-9 anos (34,7%) à >80 anos (2,5%)			14,10%	**1,10%**	
Danquah et al.^(75)^	Haiti	2012	3.132 indivíduos 0-16 anos (21%) a >16 anos (79%)			1,4%	**0,20%**	**0,04%**
Islam et al.^(76)^	Bangladesh	2012	3.104 indivíduos 30-35 anos (7,7%) a > 65 anos (14,7%)	**16,50%**				
				12.6% M				
				18,5% F				
Wandera et al.^(71)^	Uganda	2010	2.628 **idosos (>50 anos)**			10,7% M	1,5%M	
						15,9% F	2,2% F	
Tareque et al.^(70)^	Bangladesh	2010	4.176 **idosos (>60 anos)**			**10,32%**	**2,16%**	**0,26%**
						9% M	2% M	0,19% M
						11,7% F	2,31% F	0,34% F
Marella et al.^(77)^	Bangladesh	2010	2.315 adultos 18 -24 anos (23,8%) a > 50 anos		**2,30%**			
Bachani et al.^(19)^	Uganda	2009	57.247 indivíduos 0-14 anos (39%) a >60 anos (6%)			2,6% M		
						3,2% F		
Loeb et al.^(78)^	Zâmbia	2006	28.010 indivíduos (todas as idades)			3,70%	**2,30%**	**0,50%**
Seager e Tamasane^(49)^	África do Sul	2005	942 adultos e 305 crianças em situação de rua. (12-73 anos). Respondido por informantes-chaves			5,9% (criança)	**3,0%(crianças)**	**0%(crianças)**
						10,4% (adulto)	**1,7%(adultos)**	**1,7%(adultos)**
EXTENDED SET OF QUESTION								
Moniruzzaman et al.^(79)^	Bangladesh	2009	37.030 indivíduos (todas as idades)				**1,00%**	**0,40%**
WG/UNICEF								
Rojas-Martínez et al.^(69)^	México	2015	5.010 crianças (2-4 anos)				**0,16%**	
							0,22%M	
							0,1% F	
Geda et al.^(52)^	Etiópia	2014	21.572 crianças (0-14 anos)				**1,94%**	
							2,14%M	
							1,71% F	

* Ponto de corte recomendado pelo *Washington Group*

Legenda: F = Feminino; M = Masculino

Estudos da presente revisão que tinham por objetivo analisar fatores associados com a incapacidade auditiva, puderam identificar, entre eles, um menor nível de desenvolvimento do país, o envelhecimento, piores condições socioeconômicas (renda familiar, emprego e concessão de benefício) e gênero^(25,28,30,42,58)^. Pessoas em situações mais vulneráveis referiram dificuldade auditiva com mais frequência^(37,46,48)^. Além disso, em questionários auto referidos, a prevalência de incapacidade auditiva foi mais reportada por mulheres em comparação com os homens^(22,35,66,70)^. A diversidade desses estudos de associação parece ratificar e fortalecer o emprego do módulo WG para mensuração e definição da variável da incapacidade auditiva em diferentes contextos.

Poucos foram os estudos que estimaram medidas de acurácia (Tabela 4). Sprunt et al.^(65)^ recomendam que a investigação da validade seja analisada separadamente para cada domínio, e que sejam contemplados os diferentes pontos de corte do grau da dificuldade. Quando analisada a pergunta da audição isoladamente, foi encontrado o valor de sensibilidade e especificidade de 67% e 88% respectivamente, para o ponto de corte em “alguma dificuldade ou pior”, e 22% e 99,6% respectivamente para “muita dificuldade ou pior”^(74)^. As medidas de sensibilidade e especificidade do módulo infantil do WG para a pergunta de audição foram próximas àquelas obtidas para o módulo curto. Cabe ressaltar que foi observada melhor acurácia quando o instrumento foi aplicado ao professor^(65)^. O estudo de Mactaggart et al.^(10)^ com população de crianças e adultos em Camarões e Índia demonstrou que o ponto de corte “muita dificuldade” perde uma grande proporção de indivíduos com deficiência, de modo geral entre os diferentes domínios, e que este padrão se acentua quando considerados os domínios de visão e audição, os quais estão menos propensos a serem auto referidos nas limitações funcionais. Assim, considerando-se o WG como um instrumento de triagem da funcionalidade a nível populacional, deve-se adotar o ponto de corte da análise com ciência das suas implicações, assim como avaliar com parcimônia os resultados obtidos com base em cada um desses dos pontos de corte.

**Tabela 4:** Distribuição dos estudos que apresentaram medidas de acurácia da incapacidade auditiva obtidas para o módulo de audição do Washington Group em comparação com Audiometria Tonal Limiar

**Estudo**	**Questionário/Instrumento**	**População/Domínio analisado**		**Sensibilidade**		**Especificidade**		**Valor Preditivo Positivo**		**Valor Preditivo Negativo**	
				Alguma dificuldade ou pior	Muita dificuldade ou pior	Alguma dificuldade ou pior	Muita dificuldade ou pior	Alguma dificuldade ou pior	Muita dificuldade ou pior	Alguma dificuldade ou pior	Muita dificuldade ou pior
**Sprunt et al.^(65)^**	WG/UNICEFPergunta audição	(N=472) Crianças de 5 -15 anos - Escolas especiais e de educação inclusiva, Fiji	a*	78%	41%	88%	97%	--	--	--	--
			b*	72%	50%	95%	99%	--	--	--	--
Loeb et al.^(78)^	Short Set Módulo Completo	(N= ﻿5751) Todas as idades - Famílias zonas rurais e urbanas, Zâmbia		--	97,7%	--	98,5%	--	--	--	--
Ferrite et al.^(74)^	Short Set Pergunta audição	(N= 3567) Todas as idades – Famílias zona rural, Camarões		67%	22%	88%	99,6%	16%	65%	99%	97%

* Questionário aplicado aos: (a) Pais; (b) Professores

## CONCLUSÃO

Os instrumentos do *Washington Group on Disability Statistics* têm sido utilizados em nível mundial para identificação da incapacidade auditiva, em especial nos países em desenvolvimento. A pequena variação entre as medidas de prevalência parece ser um fator favorável à comparabilidade internacional das estimativas, uma das suas principais potencialidades.

Entretanto, observou-se nesta revisão que o ponto de corte e outras divergências metodológicas podem comprometer a comparabilidade das estimativas, e por isso recomenda-se o uso do instrumento no formato proposto pelo WG e, sempre que possível, a apresentação dos resultados considerando os dois pontos de corte mais comuns: “muita dificuldade” ou pior; “alguma dificuldade” ou pior. Mais estudos de validade do instrumento também são necessários, em especial conduzidos para investigar os diferentes pontos de corte.

Os pontos positivos e as vantagens na utilização do instrumento para mensurar a incapacidade auditiva, como a facilidade de aplicação e compreensão, permitiram a sua disseminação para uso em diferentes investigações científicas, para além dos censos e estudos de prevalência.

## Apêndice 1 Estratégia de busca utilizada de acordo com a base de dados

**Table:**

**PUBMED**	**SCOPUS**	**SCIENCE DIRECT**	**WEB OF SCIENCE**	**LILACS**
Search (((“washington group”) OR (((“short set” OR “extended set”)) AND question))) AND (((((“washington group”) OR (((“short set” OR “extended set”)) AND question)) OR (((“washington questionnaire”) OR module disability) OR “do you have difficulty”))) AND (((((((((domain) OR function) OR disability) OR impairment) OR disorder) OR “health survey”[MeSH Terms]) OR hearing) OR “hearing loss”) OR difficulty))	((TITLE-ABSKEY (“washington group”)) OR (((TITLE-ABS KEY (“short set”) OR TITLE-ABS-KEY (“extended set”))) AND (TITLE-ABS-KEY (question))) OR ((TITLE-ABS-KEY (“Washington question”)OR TITLE-ABS-KEY (“Washington module disability”) OR TITLE-ABS-KEY ({do you have difficulty})))) AND ((TITLE-ABS KEY (function) OR TITLE-ABS-KEY (domain) OR TITLE-ABS-KEY (disability) OR TITLE-ABS-KEY (impairment) OR TITLE-ABS-KEY (disorder) OR TITLE-ABS-KEY (“health survey”) OR TITLE-ABS-KEY (hearing) OR TITLE-ABS-KEY (“hearing loss”) OR TITLE-ABS-KEY (difficulty))	(((“washington group W/2 disability”) or (“washington group”)) OR ((({short set?}) or ({extended set?})) AND ({question?}))) AND (((hearing) OR (“hearing loss”) OR (difficulty)) AND ((function) OR (domain) OR (disability) OR (impairment) OR (disorder)))	Tópico: (function) *OR* Tópico: (domain) *OR* Tópico: (difficulty) *OR* Tópico: (hearing) *OR* Tópico: (“hearing loss”) *OR* Tópico: (“health survey”) *OR* Tópico: (impairment) *OR* Tópico: (disorder*) *OR* Tópico: (disability)*AND* Tópico: (“Washington question*”) *OR* Tópico: (“do you have difficulty”) *OR* Tópico: (“washington module”) *OR* (“short set” OR “extended set”) *OR* (“Washington group”)	(tw:(“washington group” OR “short set” OR “extended set”)) AND (tw:((tw:(domain)) OR (tw:(function)) OR (tw:(disability)) OR (tw:(impairment)) OR (tw:(disorder)) OR (mh:(“health survey”)) OR (tw:(hearing)) OR (tw:(“hearing loss”)) OR (tw:(difficulty))))
